# A Co‐Produced Health Literacy Programme for Pregnant Women Whose First Language Is Not English: A Qualitative Study of Women's Experiences

**DOI:** 10.1111/hex.70714

**Published:** 2026-06-15

**Authors:** Madeleine Benton, Ngawai Moss, Ruksana Begum, Gemma Cartwright, Phoebe Baxendale‐Smith, Arti Dave, Mandeep Kaler

**Affiliations:** ^1^ Department of Psychological Medicine, Institute of Psychiatry, Psychology and Neuroscience King's College London London UK; ^2^ Elly Charity London UK; ^3^ Katie's Team, Patient and Public Involvement Advisory Group London UK; ^4^ The Royal London Hospital London UK; ^5^ Queen Mary University of London London UK

**Keywords:** health literacy, maternal health, pregnancy

## Abstract

**Introduction:**

Health literacy, the ability to access, understand and use health information to make informed decisions, is a key determinant of maternal and infant health. In the United Kingdom, approximately one‐third of the 600,000 babies born annually are to mothers born overseas, many of whom have limited English proficiency. This language barrier contributes significantly to health disparities. To address this, we co‐produced a 6‐week health literacy course for pregnant women with limited English proficiency in East London. Designed to empower participants, the course aimed to improve their ability to navigate health information and services. Multidisciplinary facilitators co‐designed and delivered sessions covering topics such as anatomy, pregnancy, childbirth, postnatal care and medical decision‐making, using illustrated materials to enhance understanding. This study aimed to explore the experiences of women who participated in the programme.

**Methods:**

Sixteen participants took part in qualitative, semi‐structured interviews conducted by a qualitative researcher and an interpreter. Data were analysed using reflexive thematic analysis.

**Results:**

Analysis resulted in three themes: (1) Reflections on programme content and delivery, (2) enhanced confidence in multiple areas of life and (3) wider social and personal benefits.

**Discussion:**

This programme represents the first UK‐based, co‐produced health literacy initiative tailored specifically for pregnant women with limited English proficiency. Participants reported improved understanding of pregnancy and increased confidence in communicating with healthcare professionals. Importantly, the programme also fostered peer support networks, which contributed to sustained well‐being. These findings underscore the potential of health literacy interventions to reduce health inequalities and improve maternal outcomes, particularly when language and community engagement are central to programme design.

**Patient or Public Contribution:**

This research was co‐created with Katie's Team, a Patient and Public Involvement (PPI) Advisory Group in women's health. The project emerged from concerns raised by pregnant women, families, birthing companions and translators about the challenges faced by non‐English speaking pregnant women in accessing and understanding healthcare.

A working group including four PPI members, a health professional and two medical students helped shape the study. PPI contributors co‐designed and facilitated focus groups with 20 recently pregnant women, informing programme content and delivery.

PPI members were actively involved in co‐developing the course content and lesson planning alongside a multidisciplinary team. Three PPI members received English for Speakers of Other Languages (ESOL) qualifications, two of whom co‐delivered the programmeas teachers.

PPI members supported participant recruitment, helped contextualise the interpretation of results and co‐authored this paper. Findings were shared with PPI contributors for review and reflection. PPI members also took part in two community events to share findings.

## 1 Introduction

Health literacy is a fundamental component of individual agency and autonomy in managing health. It is defined as the knowledge, motivation and ability to access, understand, appraise and apply health information in ways that promote and maintain good health across the life course [1]. Higher levels of health literacy (i.e., meaning stronger abilities across the four core dimensions described by Sørensen et al. [1]) are consistently associated with improved health outcomes, including enhanced engagement in preventive care, better medication adherence and reduced anxiety related to health management [2, 3, 4]. Moreover, promoting health literacy supports ethical principles of autonomy by enabling individuals to participate actively in healthcare decision‐making [5]. In contrast, limited health literacy is linked to increased use of emergency services, lower uptake of preventive interventions, higher rates of hospitalisation and increased mortality [6].

Health literacy plays a particularly critical role during the perinatal period—encompassing pregnancy, childbirth and the postpartum phase—as it directly affects both maternal and infant health outcomes. Evidence from a systematic review demonstrates that low maternal health literacy during pregnancy is associated with increased risk of gestational diabetes mellitus, low birth weight and heightened maternal stress [7]. Pregnancy is a critical, teachable moment for developing health literacy. It is a period that represents a uniquely opportune moment for developing health literacy, as women are typically more engaged with healthcare services during this time due to frequent medical appointments, complex care pathways and the necessity of making informed decisions for both maternal and foetal well‐being [8]. Strengthening health literacy during this period not only facilitates informed decision‐making in the immediate term but also equips women with lifelong skills to navigate health systems, for themselves and their children, thereby contributing to improved population health outcomes [8, 9].

Women in the perinatal period who have limited English proficiency may face compounded barriers, particularly in healthcare systems where communication, written materials and services are primarily produced and delivered in English. This can hinder their ability to understand their medical options, navigate services, comprehend medical information and access essential care [10, 11, 12]. These challenges can have negative implications for maternal and infant health [13], as highlighted in the 2024 *Mothers and Babies: Reducing Risk through Audits and Confidential Enquiries* (MBRRACE‐UK) report, which aims to support the delivery of safe, equitable maternal healthcare. The report notes that 4% of women who died during or shortly after pregnancy between 2020 and 2022 were unable to speak or understand English [11]. Comparable mortality figures for fluent English‐speaking women are not reported in MBRRACE‐UK; however, the report and wider UK evidence highlight that language barriers are associated with higher rates of late booking, reduced screening uptake, delays in care and missed opportunities for intervention [11, 14]. This finding is especially concerning given that 51% of migrants in the UK do not speak English as their primary language at home [10], and that in 2023, 31.8% of the 600,000 live births in the UK were to mothers born outside the UK [15]. Targeted health literacy interventions that are culturally and linguistically responsive are therefore essential to reducing inequalities in maternal and infant health [16].

Despite the growing need, a recent systematic review identified only three studies explicitly targeting health literacy improvement in pregnant migrant populations [17]. In Denmark, an intervention combined midwife training with multilingual patient resources, including a leaflet and smartphone app. While participants reported improved awareness of pregnancy warning signs, there was no significant change in overall health literacy [18]. In Australia, an intervention using posters and animated videos aimed to improve maternal health literacy among Arabic‐ and Dari‐speaking women, but no published evaluation of its effectiveness is available [19]. Another Australian study piloted culturally adapted new‐parent classes for Bengali and Mandarin‐speaking mothers, resulting in improved health literacy and greater provider awareness of access barriers [20]. These limited and varied approaches highlight both the potential and the gap in evidence‐based, linguistically tailored perinatal health literacy interventions.

Given the persistent disparities linked to language barriers in UK maternity care, there is a critical need for health literacy interventions. This study offers insight into an alternative, co‐produced model designed around the communities they aim to support. It explores the experiences of Bengali (Sylheti) speaking women who participated in a 6‐week health literacy course in East London. Developed in collaboration with a local East London women's health charity, the course aimed to enhance perinatal knowledge, support English language development, and build confidence in navigating healthcare settings. As maternal populations become increasingly diverse, healthcare systems must adapt to address linguistic and cultural barriers. This intervention represents one such effort to empower pregnant women through health literacy, supporting more active engagement in their care. This study aimed to explore women's experiences of participating in a co‐produced health literacy programme, including their perceptions of how the programme influenced their understanding of pregnancy, confidence and interactions with healthcare services.

## 2 Methods

### 2.1 Study Design

A qualitative study design incorporating individual semi‐structured interviews was employed to explore participants' experiences of the 6‐week health literacy course. All individuals recruited for the study self‐identified as women and mothers; accordingly, they are referred to as such throughout. This study was reported in accordance with the Consolidated Criteria for Reporting Qualitative Research (COREQ) (Supporting Information: Appendix 1).

### 2.2 The Health Literacy Programme

A multidisciplinary team (including health professionals, ESOL teachers, PPI contributors, birth partners who support non‐English speaking women, visual designers and Charity representatives) co‐produced this health literacy programme, tailoring content towards pregnancy, childbirth and motherhood aligned with health literacy principles.

Prior to programme development, two focus groups were conducted with pregnant and postnatal women living in East London (*n* = 20, *n* = 23) with limited English proficiency. These groups, held in Bengali (Sylheti) with interpreter support, explored women's priorities for the course content, preferred formats and the acceptability of combining English language learning with perinatal content. This approach ensured that women's priorities guided the programme content, while implementation details were co‐produced with those who work closely with pregnant women who do not speak English. Following these focus groups, the health literacy programme was developed within six workshops held over a 12‐month period. Workshops were held with health professionals (midwives, obstetricians, psychologists, nurses), interpreters for the NHS, ESOL teachers, community researchers, charity organisations, women with lived experiences, all guided by an advisory group of women of Bangladeshi background.

The programme was informed by established health literacy principles and frameworks, particularly the integrated model of health literacy and the functional, interactive and critical health literacy domains [1]. In line with these principles, the programme was designed to support women in: (1) accessing health information (e.g., understanding how to navigate maternity services and appointments); (2) understanding health information (e.g., use of visual materials, simplified language and bilingual delivery); (3) appraising information (e.g., discussing symptoms, risks and decision‐making in pregnancy); and (4) applying knowledge in real‐world contexts (e.g., role‐play scenarios and communication with healthcare professionals). The inclusion of interactive teaching methods, visual aids and opportunities for communication practice was intended to move beyond functional knowledge acquisition and support the development of confidence, autonomy and engagement with healthcare services [1].

**Figure 1:**
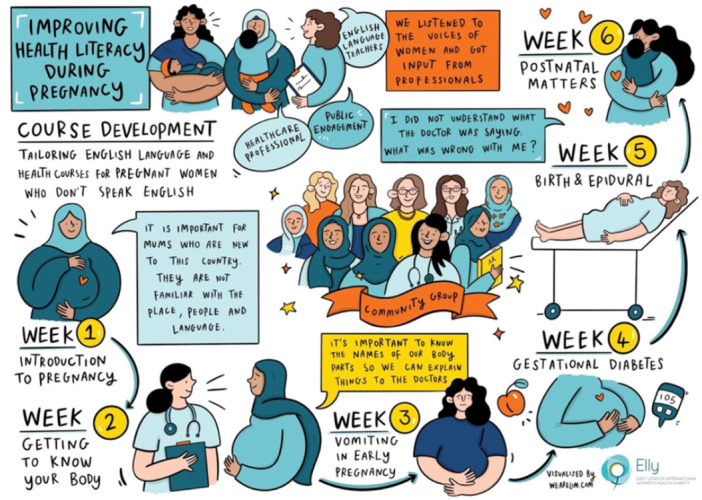
Summary of week‐by‐week content using programme illustrations created by Lim.

The health literacy programme included weekly 2‐h sessions that covered various areas as outlined in Table 1. The week‐by‐week content is showcased below using illustrations designed exclusively for the programme (Figure 1). Before the 6‐week programme, there was also an introductory session to welcome the women and introduce the aims and objectives of the programme.

**Table 1:** Summary of the health literacy programme outline, objectives and topics.

Session	Content objectives	Topics
Week 1: Introduction to Pregnancy	Understand basic health terminology, concepts and the roles of healthcare professionals.	Stages of Pregnancy, Common Health Issues, Introduction to Healthcare Professionals, Key Vocabulary.
Week 2: Getting to know your Body	Basic understanding of the female body and how it changes during pregnancy	Anatomy, Reproductive Health, Body Changes During Pregnancy.
Week 3: Nausea and Vomiting	Understand physical changes, potential complications during pregnancy, treatment options, and what to expect if hospital care is needed.	Nausea, Vomiting in Pregnancy, Hyperemesis Gravidarum and Managing Symptoms.
Week 4: Gestational Diabetes Mellitus (GDM)	Understanding gestational diabetes and its impact on pregnancy.	Causes, Diagnosis, Management, Risks to Mother and Baby, the role of Diet and Exercise.
Week 5: Labour and Childbirth	Understanding the childbirth process, modes of labour, Induction of Labour (IOL) and pain relief options.	Labour and Childbirth, IOL Reasons and Procedures, pain relief, modes of birth, warning signs, healthcare team roles.
Week 6: Postnatal Care	Understanding common postnatal experiences and symptoms, how to care for yourself physically and emotionally, and when or where to seek help.	Mental Health, Postnatal Depression, Breastfeeding, Bleeding, Bladder, Bowel and Wound Care, Pelvic floor exercises and self‐care support.

### 2.3 Programme Delivery

The programme was delivered by two ESOL (English for Speakers of Other Languages) teachers, supported by healthcare professionals (Obstetricians) and a Bengali (Sylheti)‐speaking community researcher. Each session included a quiz to reinforce learning from the previous week, and PowerPoint slides were used to guide instruction. The sessions were interactive and involved small group activities to aid participant learning. Facilitators collaborated with illustrators to develop visually engaging materials and a take‐home programme booklet (Figure 1). This interdisciplinary, culturally sensitive approach ensured the programme addressed the diverse needs of participants. The programme was piloted in a community setting to test delivery. Teaching methods were refined, but the content remained unchanged.

### 2.4 Setting

This study was conducted in Tower Hamlets, London, where 69% of the population identify as belonging to an ethnic minority group [21]. Nearly half of the female population is of childbearing age, and approximately 80% of the 5000 annual births at the Royal London Hospital are to women of South Asian descent [22]. A significant proportion of these women have limited English proficiency and primarily speak Bengali (Sylheti), creating communication barriers that hinder effective pregnancy management in an English‐speaking healthcare system.

### 2.5 Recruitment

Participants were recruited from the antenatal clinic at The Royal London Hospital, Barts Health, in East London. Inclusion criteria were being currently pregnant, not speaking fluent English, aged over 18 and able to give informed consent. Three bilingual PPI members led recruitment, two of whom were ESOL teachers (one also a qualified translator), and the third a qualified translator. To confirm eligibility, recruiters first engaged women initially in English and then in Bengali (Sylheti) to assess comprehension and whether an NHS interpreter would ordinarily be required. This approach enabled consistent identification of women with no or very limited English.

Written recruitment and consent materials were provided in English. However, all study information, including the purpose of the study and consent procedures, was explained verbally in Bengali (Sylheti) by the bilingual PPI members to ensure participants fully understood the study before providing consent.

This approach was informed by findings from the preparatory focus groups, which indicated that written translation into Bengali (Sylheti) would not have been helpful for many participants, as while they could speak Bengali (Sylheti), they were often unable to read it and were more accustomed to verbal or phone‐based interpretation. We did not formally assess language comprehension.

All three recruiters later supported or delivered the programme.

Recruitment also involved several practical and social challenges. In some cases, spousal permission influenced participation. When this arose, the Bengali (Sylheti)‐speaking PPI members spoke with women's husbands to explain the study and what participation would involve. Not all husbands agreed, but most were supportive once the purpose and structure of the programme were understood, and several actively encouraged participation. Both women and husbands were reassured that taking part would not create an additional financial burden, as travel expenses were reimbursed, this helped facilitate attendance. Given the high level of deprivation in the local area, this was an implementation decision rather than an interview finding.

Work commitments did not limit participation, as most women were not in paid employment, and those who were, held shift‐based roles that they were able to adjust to attend.

The programme took place at a local community hall in East London near The Royal London Hospital. To support women unfamiliar with the venue, community researchers met them at two agreed transport points during the first week and accompanied them to the hall. Participants were reimbursed for travel expenses on the day, using a simple flat‐rate system of £10 (based on standard bus/tube fares) because receipts were difficult to obtain; women signed a receipt confirming payment on departure.

Continuity between recruitment and programme delivery by the same Bengali (Sylheti)‐speaking PPI members helped build trust and support attendance.

## 3 Ethical Approval

Research data and participant‐related information were managed in accordance with relevant regulatory approvals. Ethical approval was granted (REC reference: 24/LO/0458) by the Joint Barts Health/QMUL Research and Development department.

### 3.1 Theoretical Perspective and Positionality

This study is situated within a post‐positivist research paradigm [23, 24], underpinned philosophically by a critical realist ontology and an objectivist epistemology. Furthermore, this study embraces principles of positionality and critical reflexivity, acknowledging that researchers' personal perspectives, social positions and inherent biases influence their interpretations of participants' narratives, particularly when the team is bringing diverse backgrounds to the project [25, 26].

### 3.2 Data Collection

At the conclusion of the programme, women who attended at least three sessions were invited for a semi‐structured interview conducted by an experienced qualitative researcher (M.B.), and supported by a Bengali (Sylheti)‐speaking community researcher and translator (R.B.). Interviews were conducted primarily in Bengali (Sylheti), with participants occasionally using English words or phrases where they felt comfortable. Real‐time interpretation between Bengali (Sylheti) and English was provided by the community researcher to ensure participants could fully express their views. This flexible, participant‐led approach to language use enabled women with limited English proficiency to engage meaningfully in the interviews while also allowing use of English where desired.

Participants determined the mode of interview, choosing either in‐person or online (via Microsoft Teams). The semi‐structured format allowed for consistent coverage of topics while enabling flexibility to explore areas of interest. All interviews were audio‐recorded with participants' consent: in‐person interviews were recorded using a digital audio recorder, and online interviews were recorded using the Microsoft Teams recording function. Participants were reimbursed £25 in cash for their time and travel costs for in‐person interviews. Interview schedule was developed collaboratively with PPI members and community researchers, drawing on insights from the preparatory focus groups and the aims of the study. Topics were identified to explore participants' experiences of the programme, including perceived impact on knowledge and confidence, practical aspects of attendance, social interactions and support, experiences of healthcare following the programme and suggestions for improvement. The interview schedule was piloted a priori through mock interviews conducted within the research team, involving the qualitative researcher and community researcher. This process allowed refinement of question wording, sequencing and prompts to ensure clarity, cultural appropriateness and relevance to participants' experiences.

The interview schedule was developed in English; however, interviews were conducted primarily in Bengali (Sylheti), with questions translated verbally in real time by the bilingual community researcher. This approach ensured accessibility for participants while maintaining flexibility in how questions were asked and explored.

The interview schedule is provided as a Supporting Information (Appendix X). Example questions included: What has changed in your understanding of your body since attending the programme?

The qualitative researcher and community researcher who were involved with the semi‐structured interviews maintained audit trails throughout the interview and analysis process. These trails were crucial for ensuring rigorous, trustworthy research by documenting the data collection and analysis steps, thereby enhancing the credibility of the findings [27]. The audit trails also facilitated reflection on each interview; for instance, after the second interview, facilitators adjusted the approach to include more prompts and probes based on participants' responses. Audio recordings were transcribed by the Bengali (Sylheti)‐speaking community researcher (R.B.), who was also involved in interpreting the interviews. Transcription involved translation from Bengali (Sylheti) into English.

### 3.3 Data Analysis

Data were analysed using reflexive thematic analysis, following Braun and Clarke's [28] six steps. First, familiarisation involved reading and reflecting on the transcribed, anonymised transcripts by three authors (P.B.S., M.B., R.B.) to develop an in‐depth understanding of the data. Initial coding was conducted by one researcher (P.B.S.), who systematically identified and labelled meaningful segments of the data. Codes were then reviewed collaboratively with the wider research team (M.B. and R.B.), and grouped into candidate themes. Differences in interpretation were discussed and explored through regular analytic meetings, allowing themes to be refined iteratively. This process emphasised reflexive dialogue and collective interpretation, rather than seeking consensus or inter‐rater reliability, in line with reflexive thematic analysis. Themes were further developed through the creation of thematic maps to ensure coherence and clear distinctions between themes. Final themes were defined and named to accurately reflect patterns across the dataset. Throughout the analysis, a researcher diary was maintained by P.B.S., M.B. and R.B. to support reflexivity and document analytic decision, enhancing transparency and rigour [28]. Analysis was led by one author (P.B.S.), supervised by an experienced qualitative researcher (M.B.), with consultation with the community researcher (R.B.). Qualitative data analysis was conducted manually using Microsoft Word.

## 4 Results

Twenty‐one women consented to participate in the programme, all were married. The mean age was 27 years (range 20–35), most were in the second trimester at the first class, and the majority were nulliparous (first‐time mothers). Table 2 presents attendance details. Table 3 outlines key participant clinical data.

**Table 2:** Participant attendance details.

Attendance characteristic	*N*
Number of attendees each week	
Week 1	18
Week 2	17
Week 3	16
Week 4	15
Week 5	11
Week 6	14
Overall programme completion	
Completed 6 weeks	7
Completed 5 weeks	5
Completed 4 weeks	2
Completed 3 weeks	1
Completed 1 or 2 weeks	4

**Table 3:** Participant clinical data.

Demographic characteristic	*N* =
Age	
Mean (years)	27
Range (years)	20–35
Week of gestation at 1st class	
Second trimester (13 to 27 weeks)	13
Third trimester (28 to 40 weeks)	3
Parity	
Nulliparous	13
Multiparous	3

A total of 16 semi‐structured interviews were completed (10 online via Microsoft Teams, and 6 in person). Interview durations ranged from 37 to 57 min (*M* = 42 min).

**Figure 2:**
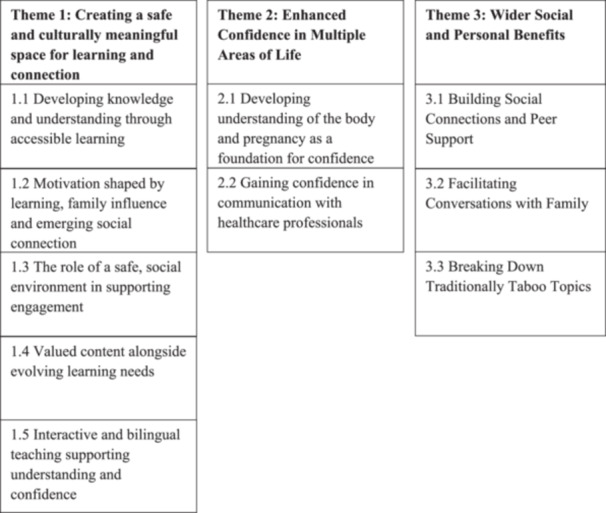
Themes and subthemes generated from reflexive thematic analysis.

Analysis resulted in the generation of three overall themes related to women's experiences of the health literacy programme (Figure 2). Quotations from the participants are presented to illustrate the themes.

Theme 1 Creating a safe and culturally meaningful space for learning and connection

*Women's accounts suggested that the programme functioned as more than a health literacy programme; it created a supportive, socially engaging and culturally meaningful environment that enabled participation, learning and confidence‐building. The combination of shared experiences, interactive teaching methods and a familiar linguistic and cultural context appeared to facilitate both knowledge acquisition and emotional well‐being. This environment also shaped women's motivations to attend and influenced how they engaged with the programme over time*.

### 4.1 Developing Knowledge and Understanding Through Accessible Learning

Women expressed high levels of satisfaction with the programme, emphasising its positive impact on their pregnancy experiences.

Coming to the course was the best decision… It was really helpful. I will carry all the information for life. It is going to help me in the future.

– P9

Women reported acquiring a comprehensive understanding of body anatomy, medical terminology, self‐care, healthcare systems and pregnancy, and highlighted how this was knowledge they had not previously possessed.

It is my first pregnancy and I am new in this country so I had very little knowledge about pregnancy and the health care system. But after doing the course I have learned so many things about pregnancy and medical terms. I got to know how to take care of myself during pregnancy and…my baby's health.

– P10

#### 4.1.1 Motivation Shaped by Learning, Family Influence and Emerging Social Connection

Women discussed how their initial motivation for attending the programme was to develop their knowledge about pregnancy and to enhance their English language skills.

I wanted to improve my English, and learn more about pregnancy as it is my first pregnancy, also I wanted to make new friends.

– P5

Many participants reported being encouraged to enrol in the programme by their husbands and extended families, highlighting the significant influence of their husbands on their decision to attend.

I will say 95% decision was from my husband. He really encouraged me to do the course.

– P5

He [husband] said I will feel better if I attend to the course and socialise with other women. He also said I can have some fresh air if I go out of the class as I don't work. So it will be good for me mentally and physically.

– P7

Conversely, some participants faced discouragement from their husbands, despite their personal desire to attend, with reasons including childcare and job‐seeking responsibilities.

He said I am pregnant so in this condition may be its not safe to go out much. Because of job centre appointment I need to go there every other week so he was more cautious for my health. He told me you even don't work so you don't need to join the course, stay at home and look after the kids. That's your duty…Then again I told him when you called and explained about the course then he said it's up to me. If I feel to go I can go as it is only for 2 h and very close to my house… I was not sure because of my husband. He was not really supportive and I don't wanted to make him upset. So I took time to make him agree

– P13

Motivation to attend the programme shifted after joining. Women were motivated by the social connections they were developing as the programme progressed. This enhanced their sense of well‐being and enjoyment.

When I came to the course and met everyone I felt so happy.

– P12

Although most participants were first‐time mothers, multiparous women also found the programme valuable, enhancing their experiential knowledge and gaining more specialised insights into pregnancy.

Even though I am a mother of two children…still I have learned so many new things in the course. I didn't know most of the body parts name in English but now I know.

– P13

#### 4.1.2 The Role of a Safe, Social Environment in Supporting Engagement

Women expressed appreciation for the environment in which the programme was delivered. They valued the in‐person format, which facilitated social interaction, supported mental well‐being and provided an opportunity to leave their homes, thereby alleviating feelings of isolation. However, a few participants indicated a willingness to participate in an online version of the programme, provided there were opportunities for engagement. This flexibility could potentially enhance attendance, particularly for those in the later stages of pregnancy.

I can talk with anyone. Pregnant woman is sitting right beside me, then I can talk with her. But online, I can't talk like I will feel shy.

– P1

It is outside of our house so change of scenario. When we go to the class maybe we take public transport or go for walk which is really good for our body and mind. When we go to the class we meet our friends which really helpful for our mental health. If you do the class online we won't able to meet anyone in person.

– P10

#### 4.1.3 Valued Content Alongside Evolving Learning Needs

Overall, women expressed high satisfaction with all content and topics within the programme. However, many identified areas for expansion on topics including the postnatal period, infant health and care and sexual health.

Add anything about baby care. We really need that.

– P9

During pregnancy how should be husband's wife's physical relationship? Till when it is safe to have sex during pregnancy? Sometimes I get scared to be intimate with my husband as I don't want to harm my unborn child. So if we get more information about it would be great.

– P10

Women also expressed a desire for more in‐depth coverage of mental well‐being during and after pregnancy, particularly regarding stress and hormonal fluctuations.

I think if you could add more information about mental health, stress during pregnancy and post‐natal then it will be really helpful for everyone.

– P8

Several women also noted that, given the timing of their attendance during the second or third trimester, the discussion of morning sickness in week three of the programme was less relevant.

First trimester…actually everyone just got throughout it so that baby care will be more helpful than first trimester.

– P1

The resources utilised in the programme, particularly the visual aids created specifically for the programme, were highly regarded by women. These resources supported both the acquisition of health‐related knowledge and the improvement of English language skills.

I found the images very useful. We are all human but we didn't know how many body parts we have or what they call…because of the clear pictures now I have learned what English words for those body parts is.

– P6

The programme booklet was also mentioned as a valuable resource, with participants using it to revisit material covered during the sessions, and to reinforce language practice outside the class. Although a few women reported feeling uncomfortable sharing the images and booklet with others, they found the materials useful for educating their husbands about the programme content.

I didn't show the booklet my husband but I always share with him my knowledge or information I have learned from the course. I think this booklet is for women and those images for our body. If they don't point out of the body part how we are going to learn? But there were two women in the class who have little children at home so they could not take out the book from their bag.

– P5

When I am alone and I don't have anything else to do I open the book and practice some English words.

– P7

#### 4.1.4 Interactive and Bilingual Teaching Supporting Understanding and Confidence

Women expressed appreciation for the diverse teaching methods employed throughout the programme. They valued the hands‐on demonstrations of medical instruments and tools. This helped to demystify healthcare practices and enhanced their understanding of healthcare autonomy and the processes involved in medical settings.

I never saw those materials [forceps] in my life and I did not know why doctor use them. Now I know what to expect when I go to labour room during my baby's birth.

– P4

The role‐playing activities were also highly regarded, as they provided valuable practice in building women's confidence and improving communication skills within healthcare contexts.

I enjoyed role plays the most…it was good practice for us to build our confidence to communicate with someone who is not Bengali.

– P10

Overall, women were satisfied with the programme's language structure. The inclusion of two teachers—one teaching in English and the other in Bengali—was particularly beneficial, creating a supportive and accessible learning environment.

I have learned many English words. I like having mixed lesson in the class English and Bengali.

– P4

Despite the success of the bilingual approach in ensuring comprehension of programme material, many participants expressed a desire for more opportunities to speak English, particularly to build their confidence and fluency.

Would love to have more interaction in English so we could improve our English more but I understood better in Bengali.

– P7

Some women suggested that including women from different language backgrounds could encourage greater use of English, as it would provide more opportunities for communication in English.

If there were women from different countries then we would force ourselves to speak English with them which is a good practice for us…the class was full of Bengali women so we spoke Bengali with each other.

– P7

In contrast, other women expressed a preference for a more homogeneous language group, feeling more comfortable when most participants spoke the same language and experienced similar cultural practices and understanding of pregnancy.

I don't mind having women from different cultures but I don't want to be the only Bengali there. So I would like to have Bengalis and other women from different countries.

– P9

Theme 2 Enhanced Confidence in Multiple Areas of Life

*Women's accounts suggested a shift from initial uncertainty and limited understanding of pregnancy and healthcare systems towards greater confidence, autonomy and active engagement in their care. Through increased knowledge of their bodies, medical terminology and healthcare processes, women described feeling more able to understand information, communicate with professionals and make informed decisions. This transition was not only cognitive but also emotional, reducing fear and enabling a greater sense of control during pregnancy*.

### 4.2 Developing Understanding of the Body and Pregnancy as a Foundation for Confidence and Autonomy

Women reported a marked increase in their health knowledge as a result of the programme, particularly regarding their understanding of their own bodies and anatomy. Women described how this new knowledge empowered them to feel more confident in their ability to navigate both pregnancy and healthcare systems, especially when having to communicate health concerns to healthcare providers.

This is my own body and I didn't know my body parts name until I got the booklet so especially for me the images were fine as I could learn more about my own body in English.

– P10

Women noted how valuable it was to be educated on topics that were previously unfamiliar to them, such as gestation periods and gestational diabetes. This greatly enhanced their understanding of pregnancy generally but also of specific health conditions too.

Now I know all things about pregnancy and I'm feeling more confident about being pregnant.

– P1

Gestational diabetes was something new for me, I didn't know much about it…the modes of labour was new for me. So week 5 was really helpful for me to learn about the normal delivery. If I would not attend to the class then I would never know what to expect in the delivery room.

– P5

Women described how this increase in health literacy had a significant impact on their autonomy and overall confidence, as they began to feel more informed about hospital systems, the roles of healthcare professionals and the various birthing options available to them. This was an important step in increasing their sense of empowerment and readiness for pregnancy and childbirth.

My confidence was very low before but now I'm more confident. Because I learnt so much about pregnancy from the course. I talk to my midwives and doctor with confidence.

– P4

I didn't know what is midwife's role actually… but after I came to the course I found out midwife look after the pregnant women and also found out about other health professional roles like health visitors.

– P10

Women discussed an enhanced understanding of culturally specific healthcare practices, which further developed their autonomy and confidence. Women explored contrasting approaches to maternity care in Bangladesh compared to in the UK.

I have learned about the steps of natural labour. But in Bangladesh first option is C‐section without trying the natural birth. So the labour modes is quite new learning for me.

– P10

In Bangladesh we were told not to carry heavy stuff or don't walk too much but in this country doctor or mid wife says you need to be physically active for a normal delivery. So the care for a pregnant women in this country is so different from Bangladesh. I am so glad I am giving birth my baby in this country. I know it will be hard as I don't have any extra hand for helping me with the baby care but at least I know me and my baby will be looked after by the NHS.

– P11

Overall, this knowledge development, enhanced confidence and increased autonomy worked together to reduce women's pregnancy‐related fears and anxieties.

I used to be scared to go to the hospital but after doing the course I am confident. I didn't know how to explain I have pain in here now I can tell I have abdominal pain.

– P9

### 4.3 Gaining Confidence in Communication With Healthcare Professionals

Women reported a notable increase in their confidence when communicating and interacting with healthcare professionals. After completing the programme, women reported feeling more capable of understanding various health conditions, articulating pain clearly and using new English terminology acquired during the programme.

Before I used to be scared with the health professionals at the hospital. I used to understand what they are saying but I didn't have enough confidence to communicate with them. But now I am more confident to communicate with them.

– P8

The programme also contributed to changes in women's perceptions of childbirth and birthing options. With a deeper understanding of these topics, they felt more informed, empowered and trusting of healthcare providers in managing their perinatal care.

In Bangladesh only one mode of childbirth which is C‐section. But in this country, I learnt from the course that they will try their best to do natural birth…it was in my mind as well maybe my baby will not born naturally. But this course has changed my thought about childbirth.

– P5

Many women expressed feeling more involved in their care, as they were better able to understand the information shared during medical appointments.

I always go with my husband. So he speaks with the doctors or midwife but now at least I understand some English words.

– P6

Theme 3 Wider Social and Personal Benefits

### 4.4 Building Social Connections and Peer Support

Women highlighted the unanticipated benefits of the programme, particularly the friendships formed with fellow attendees. Many women said they continue to stay in touch after the programme, fostering lasting well‐being impacts beyond the programme. One such significant outcome was the reduction of loneliness and isolation, which was alleviated through these new connections.

We become best friends. Sometimes we call each other and talk about our daily life. She is stressed out because she has gestational diabetes so I advise her to stay calm and look after herself. This is how we support each other. I live in my own place. When my husband goes to work I don't have anyone else to talk with. So when I'm lonely I call my friends and talk to them. We share our everyday stories or talk about our feelings. So I would say definitely making friends in the course is very impactful in my experiences. My family lives in Bangladesh, I can't call them whenever I want to but I got friends now who I can call anytime I want to as our journey is same.

– P5

Peer support also played a vital role in enhancing women's learning, as sharing personal experiences deepened their understanding of the programme material and strengthened their emotional support networks.

I learned so much from the course but I have learned from other women too. They were sharing their own personal experience about pregnancy which I felt much related with me. So it was really great to be in the same room with other pregnant women.

– P6

### 4.5 Facilitating Conversation With Family

The impact of the programme extended beyond the individual women, as many actively shared programme content with their husbands and other family members. They utilised resources such as the programme booklet to educate those not directly involved in the programme.

I have shared the booklet with my husband. He asked me lots of questions. I opened the book to show him…he learned some stuff about pregnancy. But he was encouraging me so much to attend to the classes so I can learn myself more about pregnancy. I told him during pregnancy what type of food I should eat because I had no idea about those things before. He told me he is really happy for me that I'm attending this course. I also shared with him my birth plans. What kind of pain relief they will offer me, and the process of child birth I have shared with him

– P4

But I shared with my husband. He always used to ask me after the class what did I learn… After every lesson at home I used to read the book with my husband. If some words I could not pronounce properly or I forgot how to say it, he would correct me.

– P6

This sharing of knowledge had positive outcomes, including reducing stress for both the women and their families, and fostering greater understanding and support within the household.

My husband used to be worried before I do the course as it is my first pregnancy, but now he sees me how confident I am. He is stress‐free now. So the course impacted positively in my life.

– P10

### 4.6 Breaking Down Traditionally Taboo Topics

Women identified the programme as an essential tool for addressing topics often considered taboo or difficult to discuss in their culture, particularly with family members. They appreciated the clear, accessible information provided on sensitive subjects such as sexual health, pregnancy complications and mental well‐being. Many women expressed that the programme facilitated a space for open dialogue, helping to reduce the stigma surrounding these issues. This newfound ability to discuss traditionally taboo issues not only empowered the women but also improved their confidence in seeking support from both their families and healthcare providers.

I'm in high weeks, then they will induce my labour, but nobody talk about me or I had no idea about this. Now I can talk with my family. I will be more confident about all this thing.

– P1

In our country we are not allowed to talk about pregnancy until you are married or you are pregnant. They think its shame and embarrassing to talk about pregnancy openly which is not correct. Everybody should be educated even before they get pregnant.

– P13

## 5 Discussion

This study explored the experiences of women attending a co‐produced 6‐week health literacy programme in East London, aimed at improving perinatal knowledge, English language skills and confidence in healthcare utilisation. Participants described gaining a clearer understanding of pregnancy, childbirth and the healthcare system; increased confidence in communicating with clinicians; and meaningful peer support that reduced isolation and improved well‐being. Women also reported greater autonomy in navigating care and sharing information within their households. Together, these findings show that linguistically responsive, community‐embedded programmes can strengthen both individual and collective health literacy during pregnancy.

Co‐produced health programmes, leveraging multidisciplinary approaches, foster cultural sensitivity, inclusivity and relevance, as reflected in participants’ satisfaction with the programme content [29, 30]. Moreover, the collaboration between programme community researcher and ESOL teachers and those from the target demographic during programme creation establishes approachability, relevancy and cultural sensitivity [29, 30], as reinforced by women's remarkable satisfaction with the programme content.

During programme development and delivery, barriers to women's attendance were important to consider. Women reported the significant impact of their husbands’ encouragement on attendance, aligning with previous findings of low rates of decision‐making authority and autonomy in pursuing healthcare in married Bengali women [31, 32]. Childcare and job‐seeking responsibilities were also cited as potential barriers to participation, highlighting the overall need to consider familial influence, level of independence and practical duties as important factors in attendance. For our programme, women's travel expenses were reimbursed to support their participation in the course and reduce financial barriers to attendance.

Despite the hesitation from a few husbands surrounding women's attendance at the programme, it was positive to learn that the illustrated programme resources were instrumental in broadening health literacy education for both the husband and woman. The women found the programme booklet helpful in educating their support networks on programme content as they returned home from each weekly session. Friends and family members can act as health literacy mediators, as they contribute their own skills and knowledge, and facilitate collaborative decision‐making [33]. It is therefore vital to consider how collective learning between participants and their support networks can amplify health literacy development to improve overall community health [9, 34]. Furthermore, picture‐based health education makes information more understandable, accessible and memorable [35, 36]. However, although the illustrations refined women's comprehension of key perinatal knowledge, image‐based resources alone are insufficient to enhance health knowledge in individuals with low health literacy. Effective teaching methods are needed together with visual aids to promote rigorous and accurate health literacy development [36].

Such effective teaching methods include interactive role‐play and the examination of medical instruments. The success of these techniques in progressing women's confidence and English language communication skills within the programme corresponds with previous literature. Role play is particularly successful in teaching medical terminology [37]; as an active learning process, it fosters longer‐lasting retention and more enjoyable, sociable learning [38]. Together, role‐play and the presentation of medical instruments are visually engaging and effective ways of improving medical knowledge and linguistic abilities, thereby reducing healthcare‐related anxiety [39, 40]. Thus, interactive practical approaches that transcend linguistic and cultural limitations empower women to navigate healthcare systems in an informed and relaxed manner and should be distinctly valued in future health literacy programmes.

The in‐person programme delivery setting also presented multiple benefits to women's learning. Shared learning experiences and co‐learning support the exchange of health knowledge between peers, amplifying any educational impacts and enriching programmes with a personal, contextualised quality [34, 41]. They also help to build cohesive, equitable learning communities [42]; as women gather advice and information from their peers and teachers on this programme, they become health literacy mediators for each other [33]. Many women outlined how important the in‐person setting was for these peer support networks to grow into friendships. Women supporting each other throughout pregnancy and motherhood is fundamental in their emotional and psychological well‐being, helping to sustain the improved quality of life that comes with enhanced health literacy [43, 44, 45]. However, exclusively in‐person teaching limits accessibility to those later in gestational stage or experiencing significant physical discomfort. It is therefore imperative to evaluate the most appropriate programme setting to encourage effective learning and social connection while retaining accessibility. Future programmes could investigate hybrid formats, allowing women to decide each week how they would like to learn.

This study aligns with existing evidence demonstrating that community‐based and culturally tailored health literacy programmes can improve maternal knowledge and engagement with maternity care amongst migrant and linguistically diverse women [9, 30]. Our participants valued the importance and accessibility of the course content, similar to previous co‐produced interventions [29]; however, our study adds further insight by highlighting how such education programmes can also strengthen health literacy within families and peer networks. Furthermore, earlier research has shown familial barriers faced by Bangladeshi women in accessing care [31, 32], our findings illustrate how interactive teaching, visual resources and culturally concordant facilitators can help mitigate these challenges.

### 5.1 Limitations

There are several methodological considerations that warrant reflection. First, the community researcher involved in programme organisation also acted as a translator during the interviews for data collection. While this helped build rapport and cultural familiarity, it raised the possibility of social desirability bias. To mitigate this, interviews were led by an independent qualitative researcher and analysed collaboratively with co‐researchers. Nevertheless, the potential influence of pre‐existing relationships should be acknowledged throughout research. A further limitation relates to the alignment of programme content with participants’ gestational stages. For instance, the session on hyperemesis gravidarum was reported not helpful, with many women already beyond the stage at which the condition is typically experienced. Recruitment delays presented a challenge in this respect, so future programmes should aim to either recruit participants at similar gestational stages or tailor content dynamically to participants’ needs. A final area for improvement is English language development. Although participants reported enhanced English proficiency, many expressed a desire for more structured opportunities to practice language skills. We did not use a standardised tool to assess health literacy, so improvements in this area could not be formally measured. There was no standardised measure of levels of English amongst the women, leading to a wide range of English language skills in the group. Future programmes should therefore allocate additional time to interactive, language‐focused activities to further support linguistic confidence, the effects of which could be accurately measured to assess productive ways of improving English language skills.

The programme was delivered with Bengali (Sylheti)‐speaking women from low‐income country backgrounds in a single urban area. Further work is needed to explore how the model could be adapted for other language groups or settings. Participation was influenced by family dynamics, with some women unable to take part without spousal permission. No formal English assessment was conducted, so it is unclear how group dynamics may differ in more mixed‐language cohorts.

## 6 Conclusion

This study demonstrates the potential of targeted, culturally sensitive health literacy interventions to support pregnant women with limited English proficiency. Participants reported significant improvements in key areas like birthing options, gestational diabetes and healthcare communication. Although women reported their English language skills improved, future programmes could benefit from additional interactive elements to better support language development. The programme also fostered increased confidence in healthcare settings and strengthened communication with healthcare professionals. Benefits extended beyond direct participants to those not directly involved in the programme, underscoring the value of engaging visual aids in sharing health information. Beyond knowledge acquisition, the programme facilitated the creation of support networks, fostering peer relationships which continued to benefit women throughout pregnancy and motherhood. By addressing traditionally taboo topics in a supportive environment, the programme encouraged open dialogue about sensitive issues. Ultimately, this health literacy intervention underscores the value of community‐led interventions in advancing equity in maternity care and provides practical insights for designing more inclusive and intersectional healthcare systems as diverse pregnant populations continue to grow.

Further research is needed to explore how this model can be adapted and scaled across different language groups and settings. Improving language skills, health communication and understanding through tailored health literacy support may help reduce disparities in maternity care for women with limited English proficiency.

## Author Contributions

**Madeleine Benton:** conceptualisation, investigation, writing – original draft, methodology, writing – review and editing, formal analysis, project administration, supervision, data curation. **Ngawai Moss:** conceptualisation, funding acquisition, writing – original draft, methodology, writing – review and editing, project administration. **Ruksana Begum:** data curation, methodology, formal analysis, project administration, writing – review and editing. **Gemma Cartwright:** project administration, writing – review and editing. **Phoebe Baxendale‐Smith:** methodology, formal analysis, data curation, writing – review and editing. **Arti Dave:** writing – review and editing, project administration. **Mandeep Kaler:** conceptualisation, investigation, funding acquisition, writing – review and editing, project administration, supervision.

## Ethics Statement

We were granted ethical approval for our study by the Health Research Authority and Health and Research Wales—Camden and Kings Cross Research Committee (REC reference: 24/LO/0458), the Joint Barts Health/QMUL Research and Development department.

## Conflicts of Interest

The authors declare no conflicts of interest.

## Supporting information

Supporting File

## Data Availability

The data for this study will be made available from the corresponding author upon reasonable request.
